# A Case-Control Study of the Association of Leptin Gene Polymorphisms with Plasma Leptin Levels and Obesity in the Kerala Population

**DOI:** 10.1155/2022/1040650

**Published:** 2022-12-28

**Authors:** Sudharmadevi K. Manju, Thottathil R. Anilkumar, G. Vysakh, Balakumaran K. Leena, Vijayalekshmi Lekshminarayan, Pradeep G. Kumar, Trivikrama K. Shenoy

**Affiliations:** ^1^Department of Biochemistry, Sree Gokulam Medical College and Research Foundation, Thiruvananthapuram 695607, Kerala, India; ^2^Division of Molecular Reproduction, Rajiv Gandhi Centre for Biotechnology, Thycaud PO, Poojappura, Thiruvananthapuram 695014, Kerala, India; ^3^Population Health and Research Institute, Medical College P.O., Thiruvananthapuram 695011, Kerala, India; ^4^Department of Applied Nutrition (Retd), Government Medical College, Thiruvananthapuram 695011, Kerala, India; ^5^Department of Gastroenterology, Sree Gokulam Medical College and Research Foundation, Thiruvananthapuram 695607, Kerala, India

## Abstract

**Background:**

Over the last few years, the importance of leptin in energy metabolism has been extensively studied in both animal models and in humans. Very few results are available on the association between human leptin gene (*LEP*) variants and obesity traits in India. We designed this study to analyse the polymorphisms in human leptin gene and the association of sequence variants with obesity among the population in Kerala, South India.

**Methods:**

In this case-control design of 148 study participants, data were collected on socioeconomic aspects and anthropometric measurements. Plasma glucose, insulin, leptin, and lipid profile were measured. Genotyping was done by automated DNA sequencing.

**Results:**

The common Single Nucleotide Polymorphism (SNP) of 5′-UTR of *LEP* − 2548G/A was found to be present in the study population with “A” variant as dominant allele. A novel synonymous mutation Thr5Thr of exon 2 of *LEP* was identified in heterozygous form in one subject with morbid obesity with hyperleptinemia. A novel missense mutation Phe17Leu was observed in two subjects with obesity in heterozygous condition. A novel missense mutation Lys36Arg in exon 2 of *LEP* was observed in one subject with abdominal obesity and decreased serum leptin level.

**Conclusion:**

*LEP* − 2548G/A at 5′-untranslated region was found to be common with the mutant “A” variant in the study population. SNPs of exons in *LEP* were found to be rare but associated with morbid obesity and altered levels of serum leptin in the study population in Kerala, India.

## 1. Introduction

Obesity, a systemic disease due to excessive and abnormal accumulation of body fat leading to adverse health effects, has become a major public health problem in India, especially in Kerala. Obesity is associated with higher rate comorbidities such as diabetes mellitus, hypertension, dyslipidemia, obstructive sleep apnoea, certain types of cancers, steatohepatitis, arthritis, polycystic ovary syndrome, and infertility [1]. Obesity is underpinned by positive energy balance driven mainly by hyperphagia arising as a consequence of increased hunger, decreased satiety, or both. Heritable factors account for approximately 70% of the cases with increased BMI in adult life [2, 3].

Leptin is a major adipokine that acts as a hormone to signal the brain to regulate the nutritional status by influencing feeding behaviour, metabolism, and energy balance. Leptin gene is located on chromosome 7q32.1, spanning approximately 20 kb with three exons separated by two introns [4]. The first intron is approximately 10.6 kb long and is located in the 5′-untranslated region, 29 bp upstream of the start codon. The coding region of *LEP* is 501 nucleotides long and is located mainly in exons two and three, which are separated by an intron of approximately 2.3 kb in size. The third exon consists of the downstream coding region and the 3′-untranslated region of the human *LEP* gene.

Over the last few years, the importance of leptin in energy metabolism has been extensively studied in both animal models as well as in humans. Several polymorphisms of *LEP* were studied in different populations for their potential association with obesity and metabolic complications [5, 6]. Among these polymorphisms, LEP−2548G/A and LEP 19G/A have been studied in detail [7, 8]. These findings were replicated across different populations, revealing contradictory findings [9, 10]. A few reports are available regarding the association between human leptin gene variants and obesity traits in India [11–13]. The state of Kerala stands next to Punjab in the second position in obesity prevalence in India. However, literature regarding the association between obesity and leptin gene polymorphisms among the predominantly Dravidian population of Kerala is very scarce. This study was designed to analyse the polymorphisms in human leptin gene and to study the association of variants with the incidence of obesity among the population in Kerala.

## 2. Methods

The study was approved by Institutional Human Ethics Committees of Sree Gokulam Medical College and Research Foundation, Thiruvananthapuram, Govt. Medical College, Thiruvananthapuram and Rajiv Gandhi Centre for Biotechnology. A detailed written informed consent was obtained from each participant before they could enter the study and the data were kept confidential. Study subjects with a BMI ≥ 25, with no history of any prior medication were considered as cases (obesity group). The subjects with BMI ≤ 24.9, which included normal and overweight individuals, were considered as controls. The study subjects were recruited prospectively. All study subjects were Malayalam speaking Dravidian population group from the state of Kerala in India. They belonged to various religions and subcommunities. Subjects with a history of drug intake affecting the study variables (BMI, blood glucose, insulin, lipid profile, and so on) were excluded from the study. The subjects under the age of 20 years, pregnant and postpartum women, those with polycystic ovarian diseases (PCODs) and thyroid diseases, and those taking oral contraceptive pills were excluded from the study. All the participants answered a life style questionnaire. Information about age, gender, race, socioeconomic factors, lifestyle habits, and family history of diseases was gathered using questionnaire.

We collected the blood samples from the study participants in the setting of the Obesity Clinic, Govt. Medical College, Thiruvananthapuram, Kerala, and Sree Gokulam Medical College and Research Foundation, Thiruvananthapuram, Kerala, for biochemical and gene sequencing analysis. ELISA experiments were done at Sree Gokulam Medical College and Research Foundation and genotyping by direct DNA sequencing at Rajiv Gandhi Centre for Biotechnology, Thiruvananthapuram. Even though 150 study subjects were selected for the study, sample size for the different SNPs that were examined differed and was fewer than that of the entire study due to variation in the availability of high quality DNA and incomplete successful genotyping.

10 ml of blood was collected by venepuncture into sterile vacutainer tubes with or without EDTA. Blood sugar and lipid profile were done on the same day of sample collection in a Siemens Dimension autoanalyzer using Flex reagent cartridges (Siemens Healthcare Diagnostics Ltd., Camberley, UK). The serum and plasma were stored in aliquots of 1 ml in a deep freezer at −80°C for future analysis of leptin [14] and insulin [15] by the ELISA method.

### 2.1. Selection of Single Nucleotide Polymorphisms (SNPs) of Leptin Gene

SNPs in leptin gene, which showed an association with obesity phenotypes, were identified by literature survey^5 −13^. For selecting the SNPs for the present study, we used data from the International HapMap Project (https://www.hapmap.org), National Centre for Biotechnology Information (https://www.ncbi.nlm.nih.gov), and Gene Cards (https://www.genecards.org). Eight SNPs were selected with *LEP* − 2548A/G as the most studied SNP located in the promoter region, 2 SNPs located at the 5′-untranslated region with a minimum Minor Allele Frequency (MAF) of 0.2, and 5 SNPs in the exons.

### 2.2. Genotyping by Direct DNA Sequencing

8 SNPs of human leptin gene (*LEP* − 2548A/G, *LEP* − 633C/T, *LEP* − 188C/A, *LEP* + 19G/A, *LEP* + 45G/A, *LEP* + 102C/T, *LEP* + 144A/C, and *LEP* + 328G/A) which showed significant association with human obesity were selected for the study based on the literature review (Table 1). PCR primers were designed using Primer 3 software and Primer BLAST software based on the known reference sequence information from NCBI GenBank. Peripheral blood mononuclear cells (PBMCs) were isolated from 2 ml of freshly collected blood collected in EDTA tubes by using Histopaque columns [16]. Genomic DNA was extracted from PBMC by the chemical extraction method [17, 18] using extraction buffer (1% SDS in 0.5 M NaCl), isopropanol, 70% ethanol, and Tris-EDTA buffer. PBMC was suspended in extraction buffer and sonicated mid-range 3 cycles of 10 s each. After centrifuging for 4 min, the supernatant was transferred to new microfuge tube, an equal volume of isopropanol was added, and the contents were mixed gently by inversion. The DNA pellet was washed with 70% ethanol. The pellet was allowed to air dry, and the DNA was dissolved in 50 *μ*l double distilled water. The concentration of DNA was measured spectrophotometrically at 260 nm using Nanodrop spectrophotometer (Thermo Fisher Scientific, Inc.), and the purity was assessed using the same by calculating the 260/280 nm ratio.

PCR was carried out individually for the eight SNPs being analysed. The primer pairs (Sigma Aldrich, Bangalore, India) used in this study are listed in Table 1. Each PCR reaction was composed of 1 *μ*g of the genomic DNA template, 10X reaction buffer containing 1.5 mM MgCl_2_, 0.2 mM dNTPs (New England Biolabs, MA, USA), 0.5 units thermostable Taq polymerase (New England Biolabs, MA, USA), and 1 *μ*M each forward and reverse primer in a 22 *μ*l reaction mixture. This was followed by direct DNA sequencing on Applied Biosystem 3730 series capillary automated sequencer (AB Applied Biosystems, CA, USA) using the Big Dye Terminator v3.1 Cycle sequencing kit. The sequencing PCR reaction was composed of 1 *μ*l primer (Forward or Reverse primer), 4 *μ*l 5X sequencing buffer, 1 *μ*l Big Dye Mix(AB Applied Biosystems, CA, USA), and 3 *μ*l template in a 20 *μ*l reaction mixture. The sequencing PCR products were precipitated using 2 ml sodium acetate buffer and washed with 70 *μ*l 100% ethanol. The tubes were kept in ice for 30 min and then centrifuged at 18000 × *g* for 20 min, and the pellet was washed with 70% ethanol. The tubes were kept in an inverted position over night before subjecting to automated DNA sequencing. The sequences were analysed by NCBI nucleotide-nucleotide BLAST (https://blast.ncbi.nlm.nih.gov/Blast.cgi). All sequence variants were determined and verified from the derived DNA sequence as well as by the visual inspection of the electropherogram. The results were analysed by NCBI BLAST sequence analysis initially and then by doing Multiple Sequence Alignment (MSA) of all samples.

### 2.3. Statistical Analysis

Data were analysed using Statistical Package for Social Sciences (SPSS) for Windows version 17.0 (SPSS Inc, Chicago, IL, USA). Allele frequencies were estimated by the gene-counting method, and the distribution of genotypes was tested for Hardy-Weinberg equilibrium and compared using 2 × 2 contingency tables. Testing for Hardy-Weinberg equilibrium and the significance of allelic and genotype distributions between the groups were performed by the chi-square test. To test an association between each SNP variant and phenotypes of adiposity and associated risk factors, the overall genotype test of association and genetic models (dominant, recessive, additive, allele, and homozygote) were computed. The association of phenotype traits of obesity with genotypes was analysed by Analysis of Variance (ANOVA). The statistical significance was defined by *P* < 0.05.

## 3. Results

**Figure 1:**
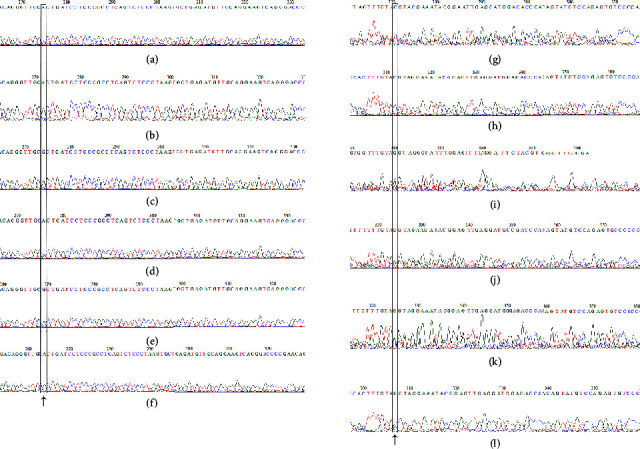
(a–f) Representative electropherograms showing −2548G/A variants in study subjects. Chromatogram showing (a) heterogenous variant (AG) in control (ID: C058); (b) homozygous variant (AA) in control (ID: C299); (c) homozygous variant (GG) in control (ID: C201); (d) heterogenous variant (AG) in case (ID: P080); (e) homozygous (AA) variant in case (ID: P151); (f) homozygous (GG) variant in case (ID: P116). Variant alleles marked. (g–l) Representative electropherogram showing *LEP* rs570757178(C/G) variants in study. (g) Homozygous (CC) in case (P293); (h) homozygous (CC) in control (C020); (i) heterozygous variant (CG) in case (P291); (j) heterozygous variant (CG) in case (P288); (k) heterozygous variant (CG) in overweight (C063); (l) heterozygous variant (CG) in subject with overweight (C155).

*LEP* − 2548A variant was found to be common in the study population in Kerala with a frequency of 66%. About 30% of control group and 33% of obesity group were homozygous with AA genotype (Table 2). A representative electropherogram showing −2548G/A variants in the study subjects is shown in Figures 1(a)–1(f). The mean serum leptin level in the obesity group with dominant homozygous AA genotype (56.6 ng/ml) was found to be high when compared with other obesity groups with AG (42.8 ng/ml) and GG (49.3 ng/ml) genotypes (Table S1). No statistically significant difference in the anthropometry measures and serum leptin was observed among the obesity groups with different genotypes (Table S1). Separate analysis by gender showed that the frequency of “A” allele was same in both genders (67%) in the study population in Kerala (Table S2). Frequency of “A” allele in both obesity and control group of men was found to be 65%, while in women, obesity group had a frequency of 69% and control group had 66%. The frequency of AA genotype in obesity group was 31% while that of control group 26%.

*LEP* rs28954080 (−633C/T) at 5′ UTR is one of the leptin gene SNPs that played significant role in obesity in different populations during literature review. However, this SNP was found to be monomorphic with C allele in our study population while it was reported to have a global minor allele frequency of *T* = 0.036. On gene sequencing analysis, a novel SNP rs570757178 at 5′UTR of *LEP* with C to G transition about 90 base pair upstream of the reported SNP rs28954080 was observed in our study. Two subjects with obesity (with identification number P291 and P288) and two overweight (C063 and C155) individuals in the study population were found to be heterozygous carriers with CG genotype (Figures 1(g)–1(l)). Low serum leptin level was observed in one obesity subject (P291) (BMI 26.3 kg/m^2^ with serum leptin 7.41 ng/ml) and one overweight subject (C155) (BMI 23.4 kg/m^2^ with serum leptin 4.36 ng/ml) (Table 3). The decrease in serum leptin levels in these two subjects was found to be statistically significant in comparison with the other heterozygotes. The WHR of these two subjects was found to be high in these two subjects, which reflects the association of this SNP with abdominal obesity. No family history of obesity was observed with these heterozygotes of *LEP* rs570757178C/G.

**Figure 2:**
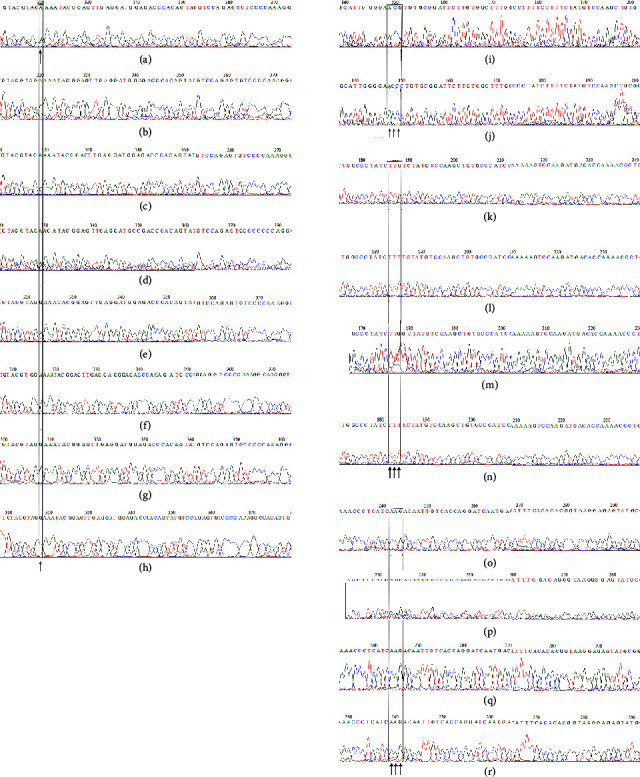
(a–h) Part of sequence chromatogram showing *LEP* rs6976701 variants in study subjects. (a) Heterozygous genotype (AG) in case (P289); (b) heterozygous genotype (AG) in obese (P286); (c) heterozygous genotype (AG) in case (P203); (d) heterozygous genotype (AG) in case (P288); (e) heterozygous genotype (AG) in case (P055); (f) homozygous genotype (AA) in case (P292); (g) homozygous genotype (GG) in control (C253); (h) homozygous genotype (GG) in case (P089). (i-j) Part of sequence chromatogram showing LEP Thr5Thr (C/G). The missense codon is marked. (i) Heterozygous (CG) in case (P184); (j) homozygous (CC) in control (C177). (k–n) A part of sequence chromatogram showing missense mutation Phe17Leu. (k) Heterozygous (GT) genotype in case (P169); (l) homozygous genotype in control (C300); (m) heterozygous (GT) genotype in case (P202); (n) homozygous genotype (TT) in case (P116). (o–r) A part of sequence chromatogram showing missense mutation Lys 36 Arg. The missense codon is shown in the box. AAG codes for Lys and AGG codes for Arg. (o) Homozygous AA in case (P055); (p) missense mutation AG (Lys 36Arg) in case (P171); (q) homozygous AA in control (C061); (r) homozygous AA in case (P288).

Another Novel SNP rs6976701 of *LEP* 5′-UTR was observed with its heterozygous genotype (AG) in five subjects with obesity (P055, P203, P286, P288, and P289) and homozygous recessive genotype (AA) in one subject with obesity (P292) (Figures 2(a)–2(h)). All other study subjects were found to have dominant homozygous GG genotype. The recessive allele “A” was found to be associated with obesity. The homozygote with recessive genotype AA was found to have strong family history of obesity. Of the five heterozygote (AG) subjects, two were observed with a strong family history of obesity (Table 3).

For *LEP* exon 1 SNP + 19G/A, no recessive homozygotes were found in the present study population (Table 2). The frequencies of homozygous dominant and heterozygous genotypes were found to be similar in both control and obesity groups. The minor allele frequency of “A” variant was found to be similar in both groups (33%). The recessive “A” variant was found to be strongly associated with adiposity measures such as BMI, hip circumference, and body fat in both control and obesity groups (Table 4). In the control group, a significant increase in waist circumference, which is a measure of abdominal adiposity, was observed in individuals with +19A variant when compared with those of +19G allele (*P*=0.002). Among the obesity group, the increase in waist to hip ratio (WHR) in individuals with mutant “A” allele is found to be statistically significant in comparison with those having “G” allele (*P*=0.001). The mean serum leptin level showed a strong association with *LEP* + 19 A variant when compared to dominant “G” allele in both control (*P*=0.01) and obesity groups (*P*=0.004).

No missense mutation at *LEP* exon 2 + 45A/G (rs145044661) was observed in the study population. Only the ancestral variant “A” is present in the study population. Homozygous dominant genotype AA was observed in all case and control subjects. However, novel mutations were observed near this SNP in exon 2 viz, Thr5Thr, Phe17Leu, and Lys36Arg.

A novel synonymous mutation Thr5Thr (rs142904532) in exon 2 of *LEP* was found to be associated with morbid obesity. In this study population, only one individual with obesity (ID number P184) had mutant allele “G” in heterozygous condition (Figures 2(i) and 2(j)). This study subject had a BMI of 44.8 kg/m^2^ and the serum leptin level of 137 ng/*µ*l (Table 3). Father and mother of this study subject were reported to have obesity.

A novel missense mutation Phe17Leu (*LEP* rs201067336) was found to be linked with obesity in the study population. In this study population, only two subjects' obesity (P169 and P202) had mutant “G” allele in heterozygous condition (Figures 2(k)–2(n)). Serum leptin levels of these two subjects were found to be abnormal (Table 3). However, no family history of obesity was reported with this subject.

The novel missense mutation Lys36Arg (*LEP* rs111650508) was observed in one subject with obesity (P171) (Figures 2(o)–2(r)), and this subject was found to be associated with abdominal obesity, decreased serum leptin level, and family history of obesity (Table 3).

**Figure 3:**
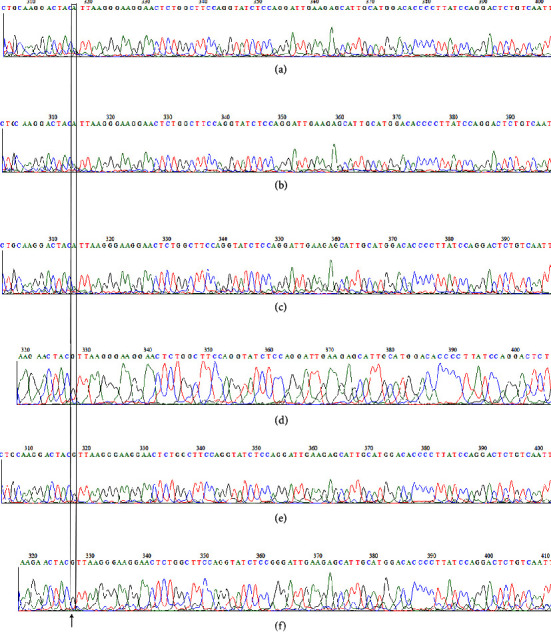
(a–f) A part of sequence chromatogram showing *LEP* + 328G/A variants. (a) Heterozygous (GA) with A variant in case (P076); (b) heterozygous (GA) with control (C145); (c) heterozygous (GA) with A variant in control C248; (d) homozygous (GG) in control (C063); (e) homozygous (GG) in case (P288); (f) homozygous (GG) in case (P298).

In this study population, no missense mutation +103C/A or +103C/G or Asn103Lys or synonymous mutation +103C/T (Asn103Asn) was observed. However, new sequence variants were observed near this region in the exon 3 of *LEP*. Analysis of rs28954114 (+328G/A) in the 3′-UTR of *LEP* revealed that the mutant allele A was observed in one subject with obesity (P076) and two control subjects (C145 and C248) in heterozygous condition (Figures 3(a)–3(f)). All other subjects had the dominant allele in homozygous form. The subject with +328A variant in heterozygous condition is reported to have family history of obesity and high serum leptin level (Table 3). The two control subjects with mutant A allele in heterozygous condition had normal serum leptin level.

## 4. Discussion

*LEP* − 2548G/A (rs7799039) is one of the most studied common SNPs of human leptin gene (*LEP*) in different populations [5, 8, 19]. *LEP* − 2548G/A is a G to A transition at nucleotide position 2548 bp upstream of the ATG start site in the 5′ promoter region of the leptin gene. In the present study, frequency of the “A” allele was found to be 66% in control group and 67% in obesity group among the population in Kerala. Li et al. reported the higher frequency of variant “A” allele in Asians at least among Malaysia/Peninsular Bumiputras, Chinese, and Indians [20]. Mammès et al. reported the strong association of G allele with overweight [21] while in our study population, the “A” allele was found to be in association with obesity. The inconsistency in various reports from different populations maybe due to different ethnicity, characteristics of subjects, or diverse covariate factors. The dominant AA genotype was found to be more common in this study population in Kerala with a frequency of 30% in control and 33% in obesity group (Table 2). The people with obesity having AA genotype showed an increase in the mean serum leptin level in comparison with AG and GG genotypes (Supplementary Table 1). This might explain the influence of the −2548A variant on the rate of transcription of leptin gene resulting in an increased level of plasma leptin. Hoffstedt et al. reported an increase in DNA binding affinity with the presence of allele A at −2548 position, which can influence leptin expression at the transcriptional level [22]. Hyperleptinemia [21, 22] and hypoleptinemia [23] have been reported in individuals carrying homozygous AA genotype by different studies. In this study population, an increase in the mean serum leptin level was observed in obesity group with dominant AA genotype (*LEP* − 2548G/A) (Supplementary Table 1). Various studies in different populations reported that there was no association of −2548G/A variants with plasma leptin levels [24, 25]. There were inconsistent reports on its effect on leptin levels with the AA genotype in Indians [6], while the G allele in Mexicans has been reported in association with hyperleptinemia [26].

Studies reported that a functional binding site for an important transcription factor involved in adipocyte differentiation named CCAAT/enhancer binding protein *α* (C/EBP-*α*) can also positively regulate the leptin gene promoter region [27, 28]. Gong et al. reported that −2548G/A polymorphism is close to a SP-1 transcription factor binding site as well as two repetitive sequences WER-11 and Alu that may regulate leptin gene transcription [29]. DNA binding affinity was found to be increased with the presence of nucleotide “A” at −2548 position, which can influence leptin expression at the transcriptional level, and thus the serum leptin level might be increased [21].

There was no association of *LEP* − 2548A variant with adiposity measures such as BMI, waist circumference, WHR, and body fat percentage. In our study population, no association of LEP-2548 A variant was observed with adiposity measures such as BMI, waist circumference, WHR, and body fat percentage. This is in agreement with Romanian and Polish subjects [30, 31]. No significant difference in serum lipoprotein levels was observed in this study population. A recent study in a Pakistani cohort reported the association of −2548A variant with HDL cholesterol level [32].

We analysed another SNP at 5′ UTR of *LEP* rs28954080 (−633C/T), which was reported to have a global minor allele frequency of *T* = 0.036. However, in our study population, these loci were found to be monomorphic with C allele. We observed a novel SNP rs570757178 at 5′UTR of *LEP* with C to G transition about 90 base pair upstream of the reported SNP rs28954080. Two individuals with obesity and two overweight individuals in the study population were heterozygous carriers with CG genotype. Low serum leptin level was observed in two individuals. Another new SNP rs6976701 was observed at 5′UTR of *LEP* with G to A transition about 85 base pair upstream of the reported SNP rs28954080. Five subjects with obesity were heterozygous carriers of GA genotype and one subject with obesity with recessive AA genotype. “A” variant was found to be associated with hyperleptinemia.

The common SNP + 19G/A (rs2167270) in the untranslated exon 1 of *LEP* showed a similar allele frequency in both obesity and control groups. The LEP A19G variant (rs2167270) is a single base transition from A to G at nucleotide position 19 in the 5′ untranslated region (UTR) of exon 1 of the LEP gene [33]. This study observed a strong association between the LEP 19A variant with obesity related variables such as BMI, waist circumference, WHR, and body fat percentage in both obesity and control groups. However, most previous studies in different populations did not find such an association [10, 24, 34, 35]. In our study population, a strong association of LEP 19A variant with serum leptin levels was observed, which is not in agreement with previous studies [10, 24].

A novel synonymous mutation rs142904532 (Thr5Thr) of exon 2 of *LEP* locates at chromosome 7 : 128252033 was observed in one subject with obesity. This mutation in heterozygous form was associated with morbid obesity with very high serum leptin level.

The missense mutation Phe17Leu (rs201067336) located on exon 2 of *LEP* (at chromosome 7:128252067) with the ancestral allele T was observed in two obese subjects in heterozygous condition. The allele T to G transition leads to missense mutation (Phe17Leu). The codon TTT codes for Phenyl alanine and TTG codes for Leucine. Of these two heterozygous individuals, one had low leptin level while the other had very high leptin level.

Novel missense mutation Lys36Arg (rs111650508) in exon 2 of *LEP* is observed in one subject with obesity in the study population. The single base substitution from A to G, resulted in change of the 36^th^ residue of leptin from Lysine to Arginine. The triplet codon AAG codes for Lysine and AGG codes for Arginine. The obese subject (BMI 26.9 kg/m^2^) with missense mutation Lys36Arg was associated with abdominal obesity with waist circumference of 104 cm and waist to hip ratio of 1.0. Serum leptin level was 6.6 *µ*g/ml. Previous studies reported that mutations in the leptin gene cause severe obesity and may also contribute to the complications associated with obesity [36, 37].

SNP rs28954114 (+328G/A) in the 3′-UTR of *LEP* was observed in one subject with obesity and two control subjects in heterozygous condition. The subject with obesity having “A” variant in heterozygous condition had increased serum leptin level with family history of obesity while the control subjects with mutant “A” allele in heterozygous condition had normal serum leptin level.

Since the *LEP* − 2548G/A region falls within the functional transcription factor binding sites, this polymorphism might affect the transcript levels of *LEP* and could account the difference in energy regulation feedback systems in different individuals. In our study, rare SNPs of mutations Thr5Thr, Phe17Leu, and Lys36Arg were detected and were found to be associated with low leptin levels and morbid obesity. Mutations of the exon regions of leptin gene generally cause leptin deficiency due to the formation of leptin protein with altered structure and thus the function due to defect in secretion, transport, and binding with specific receptors in target tissues [38]. The change in amino acid (missense mutation) during synthesis results in drastic reduction in the biological activity of the mutant leptin protein. Such mutations could play a significant role in the development of morbid obesity. The detection of such mutations in those subjects with strong family history of obesity or those subjects who are more prone to be obese could be helpful to indicate the genetic predisposition to obesity so that the incidence of obesity can be prevented or delayed by proper lifestyle modifications such as increasing the physical activity and diet control.

Increased prevalence of obesity seems to be a consequence of modern life style habits with access to excess calorie rich food plus limited physical activity. However, with the same environment, individuals respond differently. Our study could account the mechanism of such different responses in gaining weight or being healthy in the same energy rich environment that this may be largely due to genetic variation between individuals. The genetic variation and environmental factors interact to cause obesity. The genetic variations could explain why are the intervention based on diet and exercise more effective for some people only. More than that detection of such SNPs in people who are more susceptible to obesity may be beneficial for delaying or preventing this condition by adopting dietary restriction and increased physical activity.

## 5. Conclusion

DNA sequencing of the leptin gene identified 3 genetic variants in the 5′ region of *LEP*; two of them, viz., −2548G/A (rs7799039) and rs6976701 were found to be associated with hyperleptinemia indicating a key role in the regulation of rate of transcription. The −2548 G/A is found to be a common polymorphism associated with obesity among the population in Kerala. Mutations in the exons of leptin gene were found to be rare but associated with morbid obesity and altered levels of serum leptin.

## Figures and Tables

**Table 1:** List of selected SNPs of Leptin gene investigated in this study and the respective primers used for amplification of the regions of interest.

Primer ID No.	SNP/db SNP ID	Region	Primers (5′—3′)	Tm	Flanking sequences
1	−2548A/G rs7799039	Promoter-1	F-TGGGCATCCCCACAGACGGA	75.4	AGGGTTGC[A/G]CTGATCCT
			R-TCACAGTGGTCCTGAGGTGACG	69.9	

2	−633C/T rs28954080	5′-UTR	F-CACGCCTGGCTGGGTTGGTT	73.5	CCAGAGAG[C/T]GTGCACTC
			R-TGCGGGGTTGGGGGTTCTGA	75.9	

3	−188C/A rs791620	Exon-1	F-TCGCTCCTACCAGCCACCCC	72.7	GCTCCTGG[A/C]GCGCCGAG
			R-GTCCTGCCTTCTGGGCACCG	73.4	

3	+19G/A rs2167270	Exon-1	F-TCGCTCCTACCAGCCACCCC	72.7	CAGCGCCA[A/G]CGGTTGCA
			R-GTCCTGCCTTCTGGGCACCG	73.4	

4	+45G/A rs145044661	Exon-2	F-TGAGGGGATGGTAGCCAGAGCA	72.0	TCAATGAC[A/G]TTTCACAC
			R-TCAGCAGGCAGAGGGGCCAA	74.6	

5	+102C/T rs28954113	Exon-3	F-GGCTCCACCCCATCCTGACCT	72.6	CTGGAGAA[A/C/G/T]CTCCGGGAT
			R-TGCTGGGTGGACCCCCTTCC	74.6	

5	+144A/G rs1800564	Exon-3	F-GGCTCCACCCCATCCTGACCT	72.6	CTTCTTCAC[A/G/T]TGCTGGCC
			R-TGCTGGGTGGACCCCCTTCC	74.6	

5	+328G/A rs28954114	Exon-3	F-GGCTCCACCCCATCCTGACCT	72.6	AGGACTAC[A/G]TTAAGGGA
			R-TGCTGGGTGGACCCCCTTCC	74.6	

**Table 2:** Genotype and allele frequencies of rs7799039 (*LEP* − 2548G/A) and rs2167270 (+19G/A) SNPs in case and control subjects.

SNP ID	Allele/genotype	Control, *n* (%)	Case, *n* (%)	*χ* ^2^	*P* value
rs7799039 − 2548 A/G	A	88(0.656)	108(0.666)	0.03	0.90
	G	46(0.434)	54(0.333)		
	AA	20(0.298)	27(0.333)	0.35	0.84
	AG	33(0.492)	36(0.444)		
	GG	14(0.208)	18(0.222)		

rs2167270 + 19G/A	G	68(0.666)	70(0.673)	0.01	0.92
	A	34(0.333)	34(0.326)		
	GG	22(0.431)	23(0.442)	0.01	0.91
	GA	29(0.569)	29(0.558)		
	AA	—	—		

Data expressed as number (frequency). *χ*^2^ estimated with 2df for genotypes and 1df for alleles.

**Table 3:** Adiposity measures and serum leptin level of subjects with *LEP* SNPs.

SNPs	ID No.	Genotype	Age/sex	BMI (kg/m^2^)	WHR	BF (%)	Leptin (ng/ml)	Family history of obesity
rs570757178	C063	CG	36/F	23.3	0.91	33.8	27.1	No
	C155	CG	41/M	23.4	1.03	29.8	4.36	No
	P288	CG	38/M	27.6	0.93	22.5	19.7	No
	P291	CG	40/M	26.3	1.01	28.1	7.41	No

rs6976701	P055	AG	35/M	28.6	1.04	27.3	40.7	No
	P203	AG	35/M	25.1	0.91	21.6	20.6	No
	P286	AG	43/M	28	0.92	26.4	15.9	Yes (both parents)
	P288	AG	27/M	27.6	0.93	22.5	19.7	Yes (both parents)
	P289	AG	51/M	35.4	0.96	36.9	25.6	No
	P292	AA	29/M	25.2	0.92	27.8	13.2	Yes (both parents)

rs142904532 Thr 5 Thr	P184	CG	54/M	44.8	1.0	54	137	Yes (both parents)

rs201067336 Phe 17 Leu	P169	GT	39/F	27.9	0.9	42	89.3	No
	P202	GT	26/M	25.2	0.9	23	1.8	No

rs111650508 Lys36Arg	P171	AG	51/M	26.9	1.0	32	6.6	Yes (mother)

rs28954114	P076	GA	25/F	27.3	0.92	35.9	46.4	Yes (mother)
	C145	GA	37/F	17.5	0.82	28.6	5.01	No
	C248	GA	22/M	16.8	0.95	9.55	2.0	No

Study subjects identification number is given with “C” or “P” as prefix. “C” represents control and “P” case (people with obesity).

**Table 4:** *LEP* + 19G/A alleles and selected study variables.

Study variable	Case (obesity group)			Control		
	Alleles		*P* value	Alleles		*P* value
	G (*n* = 72)	A (*n* = 32)		G (*n* = 58)	A (*n* = 44)	
Age (yrs)	43.8 ± 1.5	42.9 ± 2.0	0.74	34.8 ± 1.9	40.1 ± 2.0	0.07
BMI (kg/m^2^)	27.8 ± 0.3	31.1 ± 0.6	0.001	21.1 ± 0.4	23.7 ± 0.2	0.001
Waist circumference (cm)	98.3 ± 1.0	100.9 ± 1.8	0.18	80.5 ± 1.7	87.9 ± 1.5	0.002
Hip circumference (cm)	102.4 ± 0.96	110.2 ± 1.5	0.001	89.4 ± 1.5	95.6 ± 1.9	0.01
WHR	0.92 ± 0.01	0.96 ± 0.01	0.001	0.9 ± 0.01	0.93 ± 0.02	0.24
BF (%)	33.2 ± 1.3	42.5 ± 1.5	0.001	25.8 ± 1.1	30.1 ± 1.8	0.03
Leptin (ng/ml)	45.96 ± 6.9	84.2 ± 11.2	0.004	9.4 ± 1.2	26.6 ± 7.5	0.01
Insulin (*µ*IU/ml)	31.8 ± 3.6	36.2 ± 5.4	0.49	17.2 ± 3.6	19.6 ± 3.6	0.64
HOMA-IR	15.6 ± 1.5	19.5 ± 2.8	0.19	10.1 ± 2.6	8.9 ± 1.5	0.7

Data are expressed as mean ± SEM analysed by one way ANOVA. *P* < 0.05 significant.

## Data Availability

All datasets and materials used and/or analysed during this study can be obtained from Sudharmadevi K. Manju upon reasonable request by emailing at manjuromel@gmail.com.

## Additional Points

We shall address the limitation such as small sample size utilizing only 8 SNPs.

## Supplementary Materials

Supplementary Materials Supplementary data are shown in two Tables as Table S1 and Table S2.
