# Supplementary material for: A Case-Control Study of the Association of Leptin Gene Polymorphisms with Plasma Leptin Levels and Obesity in the Kerala Population
Source: J Obes. 2022 Dec 28;2022:1040650. doi: 10.1155/2022/1040650 (PMC9812639; doi:10.1155/2022/1040650)
Supplement: Supplementary Materials — Supplementary data are shown in two Tables as Table S1 and Table S2. [file 1040650.f1.docx]

Supplementary Tables

Table S1: *LEP* -2548G/A genotypes in obesity group and selected study variables

| Study variable | Genotypes | | | P value |
| --- | --- | --- | --- | --- |
|  | AA(n= 27 ) | AG(n=36) | GG(n=18) |  |
| Age(yrs) | 43.5 ± 2.2 | 42.12 ± 1.6 | 40.4 ± 1.8 | 0.54 |
| Weight(kg) | 70.9 ± 1.5 | 73.4 ± 1.4 | 73 ± 2.06 | 0.84 |
| BMI (kg/m^2^) | 27.9 ± 0.5 | 27.7 ± 0.36 | 28.9 ± 0.6 | 0.25 |
| Waist circumference(cm) | 97.2 ± 1.3 | 97.0 ± 1.2 | 97.3 ± 1.9 | 0.96 |
| Hip circumference(cm) | 102.5 ± 1.2 | 103.1 ± 1.1 | 104.8 ± 1.8 | 0.50 |
| WHR | 0.95 ± 0.01 | 0.94 ± 0.01 | 0.93 ± 0.01 | 0.31 |
| BF(%) | 34.5 ± 1.8 | 33.5 ± 1.5 | 34.8 ± 1.9 | 0.88 |
| Leptin (ng/ml) | 56.6 ± 9 | 42.8 ± 7 | 49.3 ± 10 | 0.33 |
| FPS(mg/dl) | 100 ± 3.05 | 109.9 ± 6.8 | 108.4 ± 9.5 | 0.37 |
| Insulin(µU/ml) | 29.9 ± 3.5 | 36.6 ± 4.0 | 26.6 ± 3.7 | 0.37 |
| Cholesterol (mg/dl) | 220.5 ± 8 | 207 ± 6.9 | 203.4 ± 7.5 | 0.27 |
| Triglycerides(mg/dl) | 116.3 ± 10.0 | 134.5± 11.3 | 126.7± 13.6 | 0.52 |
| HDL cholesterol(mg/dl) | 47.9 ± 2.3 | 48.3 ± 2.4 | 49 ± 2.7 | 0.97 |
| LDL cholesterol(mg/dl) | 144.4 ± 9 | 131.3 ± 6.8 | 128.9 ± 7.2 | 0.31 |

Data expressed as mean±SEM, analysed by ANOVA

#### Table S2: Genotype and allele frequencies of *LEP* -2548G/A in the study groups according to gender

| Study groups |  | Genotypes | | | P value | Alleles | | P value |
| --- | --- | --- | --- | --- | --- | --- | --- | --- |
|  |  | AA | AG | GG |  | A | G |  |
| Male | Total  (n=78) | 27(0.346) | 36(0.461) | 15(0.192) |  | 104(0.666) | 52(0.333) |  |
|  | Case (n=46) | 16(0.347) | 21(0.456) | 9(0.195) | 0.99 | 60(0.652) | 32(0.347) | 0.96 |
|  | Control (n=32) | 11(0.343) | 15(0.468) | 6(0.187) |  | 42(0.656) | 22(0.343) |  |
| Female | Total  (n=70) | 20(0.285) | 33(0.471) | 17(0.242) |  | 94(0.671) | 46(0.328) |  |
|  | Case (n=35) | 11(0.314) | 15(0.428) | 9(0.257) | 0.77 | 48(0.685) | 22(0.314) | 0.82 |
|  | Control (n=35) | 9(0.257) | 18(0.514) | 8(0.228) |  | 46(0.657) | 24(0.342) |  |

Data expressed as number(frequency). χ^2^ estimated with 2df for genotypes and 1df for alleles.
